# Association of silent hypoglycemia with cardiac events in non-diabetic subjects with acute myocardial infarction undergoing primary percutaneous coronary interventions

**DOI:** 10.1186/s12872-016-0245-z

**Published:** 2016-04-26

**Authors:** Jian-wei Zhang, Yu-jie Zhou

**Affiliations:** Department of Cardiology, Beijing Anzhen Hospital, Capital Medical University, Beijing Institute of Heart Lung and Blood Vessel Disease, the Key Laboratory of Remodeling-related Cardiovascular Disease, Ministry of Education, Beijing, 100029 China

**Keywords:** ST-segment elevation myocardial infarction, Primary percutaneous coronary interventions, Continuous glucose monitoring system, Silent hypoglycemia

## Abstract

**Background:**

Some studies have shown that hypoglycemic episodes in diabetic patients might be associated with increased cardiovascular events. It is not clear whether episodes of silent hypoglycemia had greater prognostic value on cardiac events compared with normoglycemia or hyperglycemia in non-diabetic patients, so the aim of this study was to investigate the association of silent hypoglycemia and cardiac events in non-diabetic patients with acute ST-segment elevation myocardial infarction (STEMI) who underwent primary percutaneous coronary intervention (p-PCI).

**Methods:**

We enrolled non-diabetic patients with STEMI who underwent p-PCI and whose clinical and laboratory data were collected. Interstitial glucose values were recorded using a continuous glucose monitoring system (CGMS), and Holter monitoring was recorded for 3 days in parallel. Cardiac ischemia and ventricular arrhythmia was evaluated.

**Results:**

Based on the inclusion and exclusion criteria, we enrolled 164 STEMI patients undergoing p-PCI for final analysis. A total of 280 episodes of silent hypoglycemia (CGMS glucose <70 mg/dl) were recorded. Episodes of silent cardiac ischemia were recorded in 50 of 280 hypoglycemic episodes. The incidence of silent cardiac ischemia during hypoglycemia was significantly higher than the incidence during both hyperglycemia and normoglycemia(*P* < 0.01). Moreover, we found a significantly higher frequency of ventricular extrasystoles (VESs) or nonsustained ventricular tachycardias (NSVTs) in patients with silent hypoglycemia. The average number of events of silent cardiac ischemia was also significantly increased in the silent hypoglycemia group (0.91 ± 0.82 vs. 0.35 ± 0.54, *P* < 0.01) compared with either hyperglycemia or normoglycemia group.

**Conclusions:**

Hypoglycemia was frequent and most of the time asymptomatic in non-diabetic patients with STEMI undergoing p-PCI. Silent hypoglycemia was associated with silent cardiac ischemia. STEMI patients with silent hypoglycemia had a significantly higher frequency of VESs or NSVTs.

## Background

Glycemic management in subjects with acute myocardial infarction (AMI) remains controversy. The general consensus in present is that hyperglycemia (>10 mmol/L) and hypoglycemia (<3.9 mmol/L) should be avoided for critically ill patients [1]. AMI patients with hypoglycemia appeared to have worse outcomes [2–4]. Pinto et al. [3] thought an admission blood glucose <4.5 mmol/L had a 3-fold increased rate of adverse outcomes in patients with ST-segment elevation myocardial infarction (STEMI). Moreover, a previous study showed that spontaneous hypoglycemia was associated with a 2-fold increased in-hospital mortality in AMI patients [4]. Many studies [5, 6] showed that hypoglycemia was common in patients with type 2 diabetes and the vast majority of hypoglycemic episodes were asymptomatic and occurred at night. Moreover, with the use of continuous glucose monitoring systems (CGMS), which could monitor hypoglycemia conveniently, by simultaneously equipping subjects with CGMS and Holter monitoring, Chow et al. [5] revealed that hypoglycemia was associated with possible ischemic changes and various cardiac arrhythmias, suggesting that these events could be interconnected.

Previous studies have focused on the prognostic effects of iatrogenic hypoglycemia in diabetic patients. However, there have been few studies examining hypoglycemia-induced arrhythmias and cardiac ischemia in non-diabetic patients with STEMI undergoing primary percutaneous coronary intervention (p-PCI). Our previous study have approved that the use of CGMS was feasible and safety in STEMI subjects undergoing p-PCI Therefore, with the use of CGMS and Holter monitoring in parallel, the aim of this study was to investigate the association of silent hypoglycemia and cardiac events in non-diabetic subjects with STEMI who underwent p-PCI.

## Methods

### Study design and patient population

This study was conducted from January 2012 to November 2013. After admission, information about previous clinical history, cardiovascular risk factors, and medication were collected. Moreover, laboratory and echocardiography data were recorded. The inclusion criteria were as follows: 1) confirmed admission diagnosis of STEMI and undergoing p-PCI; 2) admission glucose <22.2 mmol/l and no history of diabetes; and 3) written informed consent. The exclusion criteria included: 1) a history of hepatic or renal impairment or of other diseases that could influence glucose metabolism,including malnutrition and cancer; and 2) a history of diabetes, cardiac pacing,or ventricular arrhythmia. The study protocol was approved by the Medical Ethics Committee of Beijing Anzhen Hospital, Beijing Daxing Hospital, Capital Medical University.

### Definition of hypoglycemia and hyperglycemia

Silent hypoglycemia was defined by no typical symptoms of hypoglycemia being noted, but the plasma glucose concentration was <3.9 mmol/l (70 mg/dl) [7].

Hyperglycemia (stress hyperglycemia) was defined as a plasma glucose concentration of at least 10 mmol/l (180 mg/dl) [8, 9].

### CGMS and holter

All of the patients were equipped with CGMS (Medtronic Mini-Med, USA) and were monitored for 72 consecutive hours after p-PCI. A CGMS sensor was inserted in the abdominal subcutis and was calibrated every 6 h according to the manufacturer’s indications. blood glucose levels of each patient was examined by a self-monitoring of blood glucose (SMBG) device (Medisafe Mini, Terumo, Japan) at least 4 times per day. The sensor measures interstitial glucose every 10 s and records the mean values at 5-min intervals. The sensor remained in place for 3 days for the collection of data, having been adapted from previously established criteria for optimal accuracy of the CGMS [10, 11].

Holter was used to monitor for cardiac ischemia and arrhythmia simultaneously. Continuous glucose and Holter monitoring was performed over a period of 72 h. The CGMS and Holter were removed after 72 h.

The Holter monitoring recordings were read by a cardiologist, and the glucose monitoring results were read by an endocrinologist, both of whom were blinded to the other’s results.

### Definitions of cardiac ischemia and ventricular arrhythmia

Silent cardiac ischemia was defined as no typical symptoms of chest pain being noted, but ischemic ECG abnormalities were recorded. Ischemic ECG abnormalities included ST-segment depression and T-wave abnormalities. ST-segment abnormalities indicative of ischemia were defined as flat or down-sloping segment depressions with ST-J depression of ≥1.0 mm in ≥2 adjacent leads. T-wave abnormalities indicative of ischemia were defined as any negative or biphasic T-wave in ≥2 contiguous leads [12].

Ventricular arrhythmia included ventricular extrasystoles (VESs), couplets, triplets, and ventricular tachycardias (VTs).

All of the cardiac event data were adjudicated by an experienced cardiovascular physician blinded to the clinical details and outcomes.

### Statistical analysis

CGM parameters were analyzed using Medtronic MiniMed CGMS software, version 3.0. The data are presented as frequencies and percentages for categorical variables and as the mean ± SD for continuous variables. We used the *χ*^2^ test to compare the categorical variables, and the 2-sample *t* test for continuous variables. Hypoglycemic and hyperglycemic episodes were compared with episodes of silent cardiac ischemia or typical angina. Hypoglycemic and hyperglycemic episodes occurring within the preceding 30 min of an ischemic event were noted [13]. Statistical analysis was performed using the Yates-corrected *χ*^2^ test. A *P* value <0.05 (two-sided) was considered significant. The data were analyzed with SPSS software, version 21.0 (Chicago, Illinois, USA).

## Results

Based on the inclusion and exclusion criteria, we enrolled 172 STEMI patients undergoing p-PCI, and six cases were excluded for final analysis due to CGMS signal interruption or failure to meet the accuracy requirements. Two cases with symptomatic hypoglycemia were also excluded. Data from the remaining 164 subjects (115 men and 49 women) were incorporated into the statistical analysis.

Table 1 provides the basic characteristics and CGMS features of the study patients. The mean number of episodes of hypoglycemia per patient was 1.7 ± 1.8, and the mean number of episodes of hyperglycemia per patient was 2.4 ± 2.7. The mean duration of hypoglycemia per patient was 1.6 ± 3.0 h, and the mean duration of hyperglycemia per patient was 2.5 ± 3.9 h.

**Table 1:** Clinical characteristics at baseline and CGMS features of study participants

Parameter	value
Subject number	164
Age (years)	53 ± 15
Males	115(70)
BMI, kg/m^2^	24.7 ± 4.1
LVEF, %	54.2 ± 7.4
HbA1c (%)	5.1 ± 1.0
Glycated albumin (%)	12.8 ± 4.6
Oral beta-blocker therapy(n,%)	135(82.3)
Risk factors (n,%)	
Hyperlipidemia	61(37.2)
Hypertension	65(39.6)
Current smoking	76(46.3)
Family history	21(13)
Obesity	35(21)
CGMS parameters	
MBG(mmol/l)	6.6 ± 0.8
MAGE (mmol/l)	2.8 ± 1.6
SDBG(mmol/l)	1.4 ± 0.5
The number of Mean episodes of hypoglycemia per patient	1.7 ± 1.8
The numberof Mean episodes of hyperglycemia per patient	2.4 ± 2.7
Mean duration of hypoglycemia per patient (h)	1.6 ± 3.0
Mean duration of hyperglycemia per patient (h)	2.5 ± 3.9
Angiographic data (n,%)	
Single vessel	71(43.3)
Double vessels	50(30.5)
Triple vessels	43(26.2)
Main stem involved	20(12)
Multivessel	93(57)

Data given as mean ± SD or n (%)

*BMI* body mass index, *LVEF* left ventricularejectionfraction, *HbA1c* Hemoglobin A1c, *MBG* Mean blood glucose, *MAGE* the mean amplitude of glycemic excursions, *SDBG* the standard deviation of blood glucose values

A total of 280 episodes of silent hypoglycemia (CGMS glucose <70 mg/dl) were recorded. Episodes with silent cardiac ischemia were recorded in 50 out of 280 hypoglycemic episodes. Episodes with typical angina were found in 23 of 280 hypoglycemic episodes (Table 2). Hyperglycemia (CGMS glucose >180 mg/dl) occurred a total of 473 times. Of these 473 episodes of hyperglycemia, 32 silent cardiac ischemia was noted, and 20 episodes of typical angina occurred. There were seven episodes of silent cardiac ischemia and five episodes of typical angina during normoglycemia (Table 2). The difference between the frequency of silent cardiac ischemia during hypoglycemia and the frequency during both hyperglycemia and normoglycemia was statistically significant (*P* < 0.01). The difference between the frequency of typical angina episodes with silent hypoglycemia and with both hyperglycemia and normoglycemia was also statistically significant (*P* < 0.01) (Table 2).

**Table 2:** CGMS and Holter monitoring abnormalities

	Total episodes	Episodes with silent cardiac ischemia	Episodes with typical angina
Silent Hypoglycemia	280	50*	23*
Normoglycemia	N/A	7 *	5*
Hyperglycemia	473	32 *	20*

*P < 0.01

Table 3 shows the relationship between silent hypoglycemia and cardiac events over 3 days of parallel recording. Three patients were excluded due to frequent malignant arrhythmia, which was treated with medication intervention or electroversion. We divided the whole study population into a silent hypoglycemia group (*n* = 55) and non-silent hypoglycemia group (*n* = 105). Mean QTc, as well as couplets per patient, triplets per patient, and SVTs (sustained ventricular tachycardias) per patient, was not different; however, we found a significantly higher frequency of VESs or NSVTs (nonsustained ventricular tachycardias) in patients with silent hypoglycemia. Moreover, the average number of silent cardiac ischemia was significantly higher in the silent hypoglycemia group (0.91 ± 0.82 vs. 0.35 ± 0.54, *P* < 0.01).

**Table 3:** Relationship between silent hypolycemia and cardiac events during 3 days of parallel recording

	Silent hypolycemia (*n* = 55)	No Silent hypolycemia (*n* = 106)	P
Silent cardiac ischemia	0.91 ± 0.82	0.35 ± 0.54	0.001
Mean QTc(ms)	391.4 ± 55.3	384.6 ± 51.7	0.441
VESs per patient(n)	4681 ± 6784	2685 ± 4378	0.048
Couplets per patient(n)	28.4 ± 58.1	20.7 ± 50.3	0.384
Triplets per patient(n)	3.4 ± 7.2	2.1 ± 6.4	0.244
NSVTs per patient(n)	2.8 ± 5.7	1.1 ± 3.5	0.043
SVTs per patient(n)	0.11 ± 0.37	0.06 ± 0.23	0.337

*VESs* ventricular extrasystoles, *VTs* ventricular tachycardias, *NSVTs* nonsustained ventricular tachycardias, *SVTs* sustained ventricular tachycardias

## Discussion

It is commonly accepted knowledge that CGMS can detect a significantly greater number of hypoglycemic episodes than repeated capillary blood glucose testing. Several studies have reported that the incidence of hypoglycemia in non-diabetic patients was rare [14–16]. However, non-diabetic hypoglycemia was common in critical care settings [17, 18]. With the use of CGMS, Chow et al. [5] found that in diabetic subjects hypoglycemia was frequently asymptomatic and may increase the risk of arrhythmias. In our study, we also found that hypoglycemia was frequent and most of the time asymptomatic in non-diabetic patients with STEMI undergoing p-PCI in the cardiac intensive care unit.

Recent studies have shown that symptomatic severe and mild hypoglycemia were associated with increased cardiovascular events [19, 20]. However, the impact of silent hypoglycemia on cardiac adverse events in AMI patients remains unclear. Based on the availability of CGMS, combined with continuous ECG monitoring, which makes it possible to examine the relationships between hypoglycemia and cardiac adverse events, Cyrus Desouza et al. [13] found that silent hypoglycemia was more likely to be associated with cardiac ischemia than normoglycemia and hyperglycemia in diabetic patients. In our study we also found that silent hypoglycemia was associated with silent cardiac ischemia. Our results indicated that AMI patients with silent hypoglycemia might be associated with poorer outcomes

Our investigations of parallel recording of CGMS and ECG revealed a high incidence of both silent hypoglycemia episodes and silent ventricular arrhythmias in non-diabetic patients with STEMI undergoing p-PCI. However, symptomatic hypoglycemia and severe symptomatic ventricular arrhythmias were rare. Previous studies [21, 22] have also shown that the incidence of serious arrhythmias was very low in AMI patients after successful early revascularization. Moreover, in our study we detected a significantly higher frequency of VESs and NSVTs in patients with silent hypoglycemia. The incidence of SVTs in non-diabetic patients with STEMI undergoing p-PCI was very low. These silent ventricular arrhythmias occurred more often at night than during daytime. Our foundings are similar to those of previous studies [5, 6].

Some potential mechanisms of which hypoglycemia might lead to myocardial ischemia and arrhythmias have been listed: increasing sympathetic activity which can induce vasoconstriction and platelet aggregation and consequently ischemia [23],a rise in some markers of endothelial dysfunction, such as VIII factor,von Willebrand factor, interleukins, cytokines levels, and increases in endothelin-1 and reactive oxygen species [24–26]; a prolongation in QT-segment,activation of the sympathoadrenal system and the production of proarrhythmogenic catecholamines, which can cause ventricular arrhythmias [23, 27, 28].

Following the use of CGMS, silent hypoglycemia could be conveniently monitored. Although the prognostic value of silent hypoglycemia in AMI patients remains controversial, it might be an important predictor of cardiac events after AMI. Further studies are needed to investigate the mechanisms, prognostic value and therapeutic strategies.

There were several limitations to this study. First, the number of patients was relatively small so that comparisons of some subgroups might be lack of power to detect significant differences for selected variables. Second, many of the AMI patients were on β-blockers, which can mask catecholamine-induced symptoms and increase episodes of silent hypoglycemia and silent ischemia. Third, the results of CGMS sometimes are unstable; moreover, this was an observational study and reflected only a possible association of silent hypoglycemia and cardiac events. Hence, the results of the present study should be interpreted with caution.

## Conclusions

Hypoglycemia was frequent and, most of the time, asymptomatic in non-diabetic patients with STEMI undergoing p-PCI. Silent hypoglycemia was associated with silent cardiac ischemia. AMI patients with silent hypoglycemia had a significantly higher frequency of VESs or NSVTs.

## Abbreviations

BMI: body mass index
CGMS: continuous glucose monitoring system
FBG: fasting blood glucose
LVEF: left ventricular ejection fraction
MACE: major adverse cardiac event
MBG: mean blood glucose
NSVTs: nonsustained ventricular tachycardias
p-PCI: primary percutaneous coronary intervention
SDBG: standard deviation of blood glucose values
SMBG: self-monitoring of blood glucose
STEMI: ST-segment elevation myocardial infarction
SVTs: sustained ventricular tachycardias
VESs: ventricular extrasystoles
VTs: ventricular tachycardias
